# How should health service organizations respond to diversity? A content analysis of six approaches

**DOI:** 10.1186/s12913-015-1159-7

**Published:** 2015-11-16

**Authors:** Conny Seeleman, Marie-Louise Essink-Bot, Karien Stronks, David Ingleby

**Affiliations:** Department of Public Health, Academic Medical Center, University of Amsterdam, P.O. Box 22700, 1100 DE Amsterdam, The Netherlands; Centre for Social Science and Global Health (SSGH), University of Amsterdam, Amsterdam, The Netherlands

**Keywords:** Equity, Diversity, Quality of care, Cultural competence, Healthcare organization, Responsiveness

## Abstract

**Background:**

Health care organizations need to be responsive to the needs of increasingly diverse patient populations. We compared the contents of six publicly available approaches to organizational responsiveness to diversity. The central questions addressed in this paper are: what are the most consistently recommended issues for health care organizations to address in order to be responsive to the needs of diverse groups that differ from the majority population? How much consensus is there between various approaches?

**Methods:**

We purposively sampled six approaches from the US, Australia and Europe and used qualitative textual analysis to categorize the content of each approach into domains (conceptually distinct topic areas) and, within each domain, into dimensions (operationalizations). The resulting classification framework was used for comparative analysis of the content of the six approaches.

**Results:**

We identified seven domains that were represented in most or all approaches: organizational commitment, empirical evidence on inequalities and needs, a competent and diverse workforce, ensuring access for all users, ensuring responsiveness in care provision, fostering patient and community participation, and actively promoting responsiveness. Variations in the operationalization of these domains related to different scopes, contexts and types of diversity. For example, approaches that focus on ethnic diversity mostly provide recommendations to handle cultural and language differences; approaches that take an intersectional approach and broaden their target population to vulnerable groups in a more general sense also pay attention to factors such as socio-economic status and gender.

**Conclusions:**

Despite differences in labeling, there is a broad consensus about what health care organizations need to do in order to be responsive to patient diversity. This opens the way to full scale implementation of organizational responsiveness in healthcare and structured evaluation of its effectiveness in improving patient outcomes.

## Background

Health service users belonging to groups that differ from the majority population, such as migrants and ethnic minorities, often receive poorer care than majority users. Variously described as health care ‘disparities’, ‘inequalities’ or ‘inequities’, these problems in health care have been well documented in the United States [1, 2] and are increasingly being recognized in other countries [3–5].

The evidence demonstrating health care inequalities implies that health services need to adapt in order to increase their accessibility and quality for minority service users. Promoting responsiveness to diversity requires interventions at individual, organizational and system level. Individual caregivers need specific knowledge, skills and attitudes [6, 7]. For health care organizations, promoting diversity responsiveness involves putting into place specific service policies and practices [8]. Diversity responsiveness at the level of entire health systems includes measures at national or state level, for example by legislation guaranteeing financial access to health care.

In the US, responsiveness to diversity at organizational level has been promoted through the CLAS standards (Standards for Culturally and Linguistically Appropriate Services) [9], designed to address the needs of racial, ethnic, and linguistic population groups that experience disparities in health service provision [10, 11]. In Europe, diversity responsiveness recommendations have been published under the heading of ‘migrant-friendliness’ [12], ‘intercultural opening’ [13], ‘transcultural competence’ [14] or ‘difference sensitivity’ [15], the latter targeting ‘vulnerable groups’ including but not confined to ethnic and cultural minority groups.

Responsiveness to ethnic and cultural diversity has for several decades been referred to as ‘cultural competence’ [16]. Regarding terminology, the term cultural competence has often been criticized in the literature [17, 18], mainly because of limited operationalizations focusing too exclusively on expert knowledge about minority populations. Critics correctly point out that such knowledge may lead to stereotyping and a focus on the ‘otherness’ of patients. However, many cultural competence frameworks have a broader operationalization, emphasizing the triad of skills, attitudes and knowledge [6].

The development of uniform terminology and definitions to describe culturally competent or diversity responsive health care is considered as key for effective evidence-based development, implementation and evaluation of diversity responsive health care [17, 19]. Therefore, we qualitatively analyzed and compared six approaches to organizational diversity responsiveness in health care. We explored whether the lack of agreement about terminology between such approaches reflects important differences at the level of content, or whether there is a reasonable degree of consensus about the measures that organizations should take to adapt health services to the needs of diverse patient populations. The paper addresses the following questions:

1. On which domains of health service provision do the approaches focus?
2. How much agreement is there about important domains?
3. What are the most consistently recommended elements for organizations to address in order to be responsive to diversity?

## Method

### Sampling strategy of approaches

In recent years many guidelines or recommendations for increasing organizational responsiveness to diversity have been published internationally by both public and private bodies. We explicitly did not aim at conducting a comprehensive review of all available approaches. Instead, we purposively sampled six approaches to diversity responsiveness at organizational level using the following criteria:

- the approach was developed for wider than local use by a single health care organization;
- it was developed by an authoritative public body, such as a health ministry or a recognized group of experts;
- it was publicly available;
- the final selection included approaches from the US, Australia and Europe.

We started with the CLAS Standards [9] and continued adding approaches one by one, searching the published and grey public literature in an iterative process, until saturation was reached (i.e. adding a new approach did not provide new information). The sampling was conducted in 2012.

The following approaches were sampled: 1) CLAS Standards - National Standards for Culturally and Linguistically Appropriate Services in Health Care (further referred to as: CLAS) developed by the Office of Minority Health, part of the U.S. Department of Health and Human Services [9]; 2) Advancing Effective Communication, Cultural Competence, and Patient- and Family Centered Care: A Roadmap for Hospitals (further referred to as JCR), developed by the US Joint Commission [20]; 3) Cultural Responsiveness Framework. Guidelines for Victorian health services (further referred to as CRF) developed by the Victorian Government, Department of Health, Australia [21]; 4) Recommendation of the committee of ministers to member states on mobility, migration and access to health care (further referred to as COER) of the Council of Europe [22, 23]; 5) The Equality Delivery System (further referred to as EDS) for the NHS, UK [24]; 6) Standards for Equity in Health Care for Migrants and other Vulnerable Groups (further referred to as EQS) developed by the WHO-HPH Task Force on Migrant-Friendly and Culturally Competent Healthcare [25]. Table 1 briefly describes each approach and clarifies our reasons for including the approach in our study.

**Table 1:** Description of the six approaches on responsive health care that were included

1. CLAS Standards - National Standards for Culturally and Linguistically Appropriate Services in Health Care (CLAS) [ 9 ]. These standards were developed by the Office of Minority Health, part of the U.S. Department of Health and Human Services. Some of the standards have the status of mandates, meaning that they are Federal requirements for all health care organizations that receive Federal funds; others are purely recommendations. We included the CLAS standards because they were the first one and probably the most comprehensive and influential approach currently in use. In May 2013 the Enhanced CLAS Standards were published [ 11 ]. Although largely similar, there are some differences of emphasis between the original and the Enhanced CLAS Standards:
• The revised CLAS acknowledged that in order to address disparities in health care (for any target group), we need to go beyond cultural issues and deal with other (e.g., social, psychological) issues.
• In the vision on responsive care some slight changes of emphasis could be found, such as a shift from regarding diversity as a ‘group’ characteristic to ‘appreciating the diversity of individuals’. The enhanced CLAS also places more emphasis on the importance of ‘patient- and family centred care’, thus bringing it more into line with the JC Roadmap.
2. Advancing Effective Communication, Cultural Competence, and Patient- and Family Centered Care: A Roadmap for Hospitals (JCR) [ 20 ]. This ‘Roadmap’ has been developed by the Joint Commission (JC), an independent, not-for-profit organization which accredits and certifies health care organizations in the United States. The Roadmap was developed in addition to existing JC requirements “to inspire hospitals to integrate concepts from the fields of communication, cultural competence, and patient- and family-centered care into their organizations.” We included the JC Roadmap because of the global influence of JC and the Joint Commission International (JCI) accreditation program on health care organizations through their accreditation programs (applied in over 50 countries). JC developed its own framework of recommendations in which cultural competence is embedded within effective communication and patient- and family centred care. Please note that 1) other existing JC requirements also include issues related to those issues discussed in the Roadmap, and 2) that the national Joint Commission Standards are different from the Standards of the Joint Commission International.
3. Cultural Responsiveness Framework. Guidelines for Victorian health services (CRF) by the Rural and Regional Health and Aged Care Services, Victorian Government, Department of Health (Australia) [ 21 ]. The CRF was developed to replace the Health Service Cultural Diversity Plans (HSCDPs) which since 2006 have required all Victorian health services to develop and implement policies for ethnic diversity in care. The intention of the CRF is to consolidate multiple requirements for reporting on cultural diversity initiatives within health services. All Victorian health services are required to submit plans and achievements according to the standards and measures in the CRF to the Statewide Quality Branch. We included the CRF because it has been disseminated and made compulsory in a large health care system in Australia.
4. Recommendation of the committee of ministers to member states on mobility, migration and access to health care (COER) of the Council of Europe [ 22 , 23 ]. The Council of Europe is an international organization set up “to achieve a greater unity between its members for the purpose of safeguarding and realizing the ideals and principles which are their common heritage and facilitating their economic and social progress” [ 37 ]. We included the COER because it has been endorsed by the Health Ministers of the 47 member states of the Council of Europe. The document is aimed at ministerial level, therefore it includes recommendations that have consequences for the whole health system. These recommendations focus on the diversity responsiveness in the context of migrants and their children. To make comparisons possible we have only included the recommendations at organizational level in our analysis.
5. Equality Delivery System (EDS) for the NHS [ 24 ]. EDS originates from the Equality and Diversity Council within the British National Health Service (NHS). It is designed to help NHS organizations to comply with the ‘Public Sector Equality Duty’ (PSED) of the Equality Act. This act “requires public bodies to consider all individuals when carrying out their day to day work – in shaping policy, in delivering services and in relation to their own employees” [ 38 ]. EDS is made available to the NHS as an optional tool. It was included because it is a European instrument which has been disseminated in a large health care system.
6. Equity Standards (EQS) of the Task Force on Migrant-Friendly and Culturally Competent Healthcare [ 25 ]. These Standards were developed by a group of mainly European experts set up within WHO’s Health Promoting Hospitals network. The Equity Standards are a self-assessment instrument to enable health care organizations to carry out an ‘equity evaluation’ against a set of standards. The instrument was piloted in 10 European countries, as well as in two non-European ones. The Equity Standards were included because of the broad international context in which they were developed.

In May 2013 the Enhanced CLAS Standards were published, entitled “National Standards for Culturally and Linguistically Appropriate Services in Health and Health Care: A Blueprint for Advancing and Sustaining CLAS Policy and Practice” [11]. A comparison of the new Standards with the original version showed that the underlying approach was virtually identical to that which informed the first edition. The aim of the revision was to increase the effectiveness of the Standards, by explaining them more clearly, ensuring that they reflected recent developments, and aligning them with other initiatives such as the Affordable Care Act and the work of the Joint Commission. Some differences of emphasis are described in Table 1. The version of CLAS used in this paper is the original one: we considered the original CLAS Standards as the oldest and most influential of all approaches and we did not feel the changes made in the enhanced version were fundamental enough to warrant a separate analysis.

### Developing an analytical framework for the present analysis

#### Encoding of content

1. An inductive approach was used. We first analyzed CLAS [9] by grouping the 14 standards into conceptually distinct topic areas (domains). We subsequently identified dimensions that showed the concrete operationalization within each domain.
2. A second approach was selected and its content was subsumed under the domains and dimensions identified in Step 1, new ones being created where necessary.
3. The remaining four approaches were treated in the same way.
4. The resulting framework of domains and dimensions was critically reviewed and discussed until consensus was reached by three of the authors in order to remove ambiguities and overlap.

#### Comparison of content

Categorizing the content of the approaches in this analytical framework enabled us to see at a glance whether certain domains were unique to, or absent from, particular approaches. Within each domain, it revealed the differences in the ways in which approaches operationalized the domain. We listed the differences between them as well as their commonalities. MCS performed the analysis; results were independently reviewed by MLE-B and JDI. Disagreements were resolved by discussion.

### Ethics statement

An ethics statement was not required for this work.

## Results

This section starts with background information on the six approaches. We then provide an overview and analysis of their content, classified according to the domains and dimensions of the analytical framework. We then describe the similarities and differences between the six approaches.

### Background information

Table 2 provides background information on the six approaches, listed with their acronyms.

**Table 2:** Background information on the six approaches

Background information on model	CLAS Standards (CLAS)	Joint Commission Roadmap (JCR)	Cultural Responsiveness Framework (CRF)	Council of Europe Recommendations (COER)	Equality Delivery System (EDS)	Equity Standards (EQS)
*origin*	US dept of Health and Human Services; Office of Minority health (U.S.)	The Joint Commission (U.S.)	Victorian Government; Dept. of Health (Australia)	Council of Europe; The committee of ministers (Europe)	The National Health Services (NHS); The Equality and Diversity Council (U.K.)	Health Promoting Hospitals; Task Force on Migrant-Friendly and Culturally Competent Health care (Europe)
*year*	2001	2010	2009	2011	2011	2013
*aim*	*ensure equitable and effective treatment in a culturally and linguistically appropriate manner *correct inequities *more responsive services *elimination of racial and ethnic health disparities *inform, guide and facilitate culturally and linguistically appropriate care	*improve overall safety and quality of care *integrate concepts from communication, cultural competence and patient-centered care fields into hospitals	*better links between access, equity, quality and safety *better health outcomes for culturally and linguistically diverse (CALD) populations *enhance cost effectiveness of service provision *track organizations' improvement; align cultural responsiveness (CR) with existing standards; develop benchmarks	*Equitable access to health care of appropriate quality	*better outcomes for patients and communities, better working environments for staff *improve equality performance *review equality performance *a tool to comply to the ‘public sector Equality Duty’.	*ensure equitable and accessible health care *reduce disparity in health care*an Equity self-assessment by health care organizations
*vision*	*cultural and linguistic competence *culturally and linguistically appropriate services (CLAS)	*effective communication (EC) *cultural competence (CC) *patient- and family-centered care (PFCC)	*cultural responsiveness (CR)	*improving the adaptation of health service provision to the needs, culture and social situation of migrants	*equality for patients and staff *personal, fair and diverse services and workplaces	*promoting equity
*target population*	*inclusive of all patients *especially racial, ethnic, and linguistic populations that experience unequal access	*no target group, recommendations address 'issues' in health care (e.g. language, culture etc.)	*Culturally and linguistically diverse populations (CALD)	*migrants	*protected groups	*migrants and all other vulnerable groups
*target organization-type*	*health care organizations *policymakers, accreditation agencies, purchasers, patients, advocates, educators, health care community in general	*hospitals	*all Victorian health services	*governments of CoE member states	*NHS commissioners and providers	*health care organizations
*structure*	*14 standards in three types: mandates (4), guidelines (9), and recommendations (1) *three themes: culturally competent care, language access services, and organizational supports for cultural competence	*54 recommendations structured around main points along the care continuum *aspects of the care continuum: admission; assessment; treatment; end of life care; discharge and transfer; organization readiness	*six standards across four domains, divided in measures and sub-measures (both quantitative and qualitative) *Standards: a whole organization approach; leadership; interpreters; inclusive practice; consumer/community involvement; staff. *Four domains: organizational effectiveness; risk management; consumer participation; effective workforce	*14 recommendations, specified in 31 sub-recommendations.	*18 outcomes grouped into four goals; nine steps for implementation *EDS goals: better health outcomes for all; improved patient access and experience; empowered, engaged, and well-supported staff; inclusive leadership at all levels	*five main standards, divided in substandards and measurable elements *main standards: equity in policy; equitable access and utilization; equitable quality of care; equity in participation; promoting equity

As their *aim*, the approaches refer to reducing or eliminating existing inequalities in health and service provision between different populations (CLAS, COER, EQS), as well as improving outcomes for individual patients (JCR, CRF, EDS). Some approaches (CLAS, COER, EQS) start from human rights principles, regarding inequalities between groups as injustices which should be eliminated. JCR considers equality of patient outcomes as a performance indicator, while CRF and EDS combine both starting points. Two unique aims also emerge: CRF aims to enhance the cost-effectiveness of health service delivery, and EDS aims to create better working environments for staff.

In their *vision* on responsive care, three approaches directly invoke the concept of ‘cultural competence’. CLAS refers to the classic definition of cultural competence (Cross et al., 1989) [16] and explicitly emphasizes language issues. For JCR, cultural competence is one of three fundamental concepts on which the Roadmap elaborates (the other concepts being ‘effective communication’ and ‘patient and family centered care’). JCR and CLAS operationalize cultural competence similarly. CRF introduces another label: ‘culturally responsive care’, but the vision implied is very similar to that of CLAS and JCR. A common characteristic of these three approaches is their emphasis on ‘culture’ in the labeling of their vision.

In the other three approaches the emphasis is not on ‘culture’ but on ‘equity’ or ‘equality’. COER refers to “equitable access to health care of appropriate quality”: in relation to service delivery, by referring to “improving the adaptation of health service provision to the needs, culture and social situation of migrants”. EDS does not provide a definition of its concept of equality, but relates it to the pursuit of quality, which in turn is defined as recognizing the needs and circumstances of *all* (both patients and staff) and ensuring accessibility, appropriateness, safety and effectiveness for patients. EQS explicitly distances itself from the concept of ‘cultural competence’, instead highlighting Whitehead’s definition of equity in health: “equal access to available care for equal need; equal utilization for equal need; equal quality of care for all” [26].

The *target population* of each approach refers to the user groups envisaged by the authors as beneficiaries. CLAS, JCR and CRF refer to the target population in terms of race, ethnicity, culture or language, while EDS and EQS also include gender, age, disability, religion, sexual orientation, transgender status (both EDS and EQS); marriage and civil partnership, pregnancy and maternity, nationality (only EDS); socio-economic status and minority status (EQS). COER focuses explicitly on migrants, a category that is not mentioned explicitly in CLAS, JCR, CRF or EDS. However, COER uses the term migrant “in a very broad sense, referring not only to those who change their country of residence voluntarily but also to asylum seekers, refugees and victims of human trafficking. Since the consequences of migration may also extend beyond the first generation, second and later generations are also discussed. In the case of Internally Displaced Persons, internal migrants are also included”. There is thus considerable overlap between the category of ‘migrants’ as defined by COER and the term ‘ethnic minorities’ used in other approaches.

## ‘Horizontal’ analysis (comparison of domain content across approaches)

### Organizational commitment

We classified elements of the various approaches in this domain if they mentioned commitment at management level to responsiveness to diversity. Two dimensions were found: *policy and leadership* and *measurement of performance*.

#### Policy and leadership

All six approaches maintain that organizations must make an explicit commitment to developing responsive care, rather than merely permitting individual initiatives that are not structurally embedded in the organization. In COER this requirement is implied by insistence on a ‘whole organization approach’. Commitment can either take the form of an explicit plan (CLAS, EQS), which sets out how the organization intends to organize and guarantee responsiveness, or a policy of good leadership (JCR, EDS), or both (CRF).

‘Good leadership’ is explicitly committed to achieving responsive care and promotes this within the organization (JCR, EDS): leaders take responsibility for reaching this goal (CRF). All approaches emphasize that plans for change should be integrated in existing organizational policies and processes. EQS additionally promotes a ‘proactive’ approach: in *all* its plans, the organization should anticipate the effect the plans will have on accessibility and quality of care for vulnerable groups.

#### Measuring and improving performance

All approaches regard it as essential that organizations measure their performance in providing responsive services (e.g. outcomes of treatment for different groups), with the aim of identifying necessary improvements, taking action, and assessing the organization’s progress in providing responsive care. CLAS, EDS and EQS further emphasize that performance measurement should be a continuous activity, incorporated in regular performance measurement systems. The approaches differ regarding the variables that should be measured: some focus on quality of care, some on accessibility and some on both (see also the domain ‘collecting data’ for the data sources to be used in measuring and improving performance). CRF is the only approach stipulating mandatory indicators for measuring organizational cultural responsiveness. These have to be submitted by affiliated organizations and are also used for benchmarking.

### Collecting data

The second domain we identified concerns the collection of data, not as an end in itself but in order to inform the organization about equity of access to and quality of care delivered in their organization and to identify special needs or opportunities for improvement. Two types of data are distinguished, concerning *the population at large* and *the organization’s own users.*

#### Data on the population at large

Five approaches (CLAS, JCR, CRF, EDS, EQS) recommend collecting data on the community or catchment area in order to adapt services to the needs thus identified. Organizations can use information that is already available, but CLAS and EDS also give organizations an active role in collecting these data themselves. Such data include demographic variables (e.g. age, gender, ethnicity), characteristics potentially affecting service use (e.g. language proficiency, health literacy), health status and exposure to health risks. In COER the importance of empirical evidence is strongly emphasized: governments are urged to collect it “in partnership with relevant organizations”.

#### Data on the patient population

Patients’ files can serve as a source of data on ethnicity, race, language and other characteristics considered relevant for quality of care. For CLAS, JCR, CRF, and EQS these data are considered important in order to identify and monitor health and health care inequalities. For CLAS, JCR and EQS, information in patients’ files on individual characteristics associated with ethnic minority status (e.g. proficiency in the majority language) also enables adaptation of care to the needs of an individual patient. Additionally CLAS, JCR, CRF, EDS and EQS emphasize that outcome measures and patient feedback systems must be able to analyze results according to diversity characteristics. The approaches differ in the types of data they recommend organizations to collect in this respect.

### Staff/Workforce

The third domain concerns the staff or workforce of the organization. Two dimensions can be distinguished: *staff competencies* and *diversity in the workforce*.

#### Staff competencies

Staff competencies in delivering responsive care, and the importance of education and training, are central themes in all approaches. CRF and EDS describe a comprehensive approach to improving confidence and competence among staff, for example through personal development programs (EDS) and adapted HRM policies (CRF). CLAS, CRF, COER and EQS emphasize that *all* staff should be trained (CLAS even includes affiliated and subcontracted staff); CLAS, CRF and EQS also recommend monitoring the effects of training. CLAS recommends separate training in the provision of responsive care; JCR recommends the incorporation of such training in the existing curriculum, while COER and EQS support both. The approaches vary in the amount of information they provide on the content of training.

#### Diversity in the workforce

According to CLAS, JCR and COER, diversity among staff members is desirable for furthering responsiveness to patient diversity. Two arguments are given for the importance of staff diversity. The first is general: the workforce should be representative of the general population (CLAS, COER). The second is more specific: staff diversity is considered to further equity by making possible a higher degree of linguistic and ethnic concordance between patients and staff (CLAS, JCR). EDS and EQS discuss this issue from the perspective of equality among staff and include objectives for inclusive Human Resource policies relating to issues such as recruitment. CRF does not address the issue of staff diversity.

### Ensuring access

Under this heading we classified all issues relating to barriers to entering the healthcare organization. The dimensions that emerged concerned *entitlement to care*, the provision of *understandable information*, and issues concerning *geographical* and *other aspects* of accessibility. Some issues discussed in this domain reappear in the domain of ‘care provision’, because they are also relevant to the caregiving relationship.

#### Entitlement to care

Entitlement to care (i.e. whether patients are insured or are allowed to use national health services) is not mentioned in CLAS, JCR, CRF and EDS. This is understandable to the extent that entitlement is an issue covered by legislation and insurance rules, rather than by the policies of service providers. If service providers choose to give care outside the framework of formal entitlements, this is left to their own discretion. However, EQS goes a step further and charges organizations with a responsibility for patients who are not eligible for care: it urges that at the very least, steps should be taken to help them find appropriate care elsewhere. COER, which includes recommendations at health system level, makes a plea for “adequate entitlements to use of health services”: concerning the role of service providers, it stresses that these must ensure that legislation is implemented properly and that all care providers are aware of existing rights.

#### ‘Understandable’ information

Three approaches (CLAS, COER and EQS) stress that organizations should provide ‘understandable’ information in order to facilitate accessibility. This means providing information which is translated where necessary and is adapted to the health literacy level of the targeted populations.

#### Geographical accessibility

The importance of reducing geographical barriers to accessibility is briefly discussed in COER and EQS.

#### Other aspects of accessibility

In EDS and EQS two unique dimensions related to accessibility appeared. Firstly, EDS mentioned specific types of care (public health, vaccination and screening programs); secondly, EQS discussed the accessibility of organizations for specific ‘disadvantaged’ target groups such as HIV/AIDS patients, disabled patients, and homeless people.

### Care provision

Issues in this domain relate to the quality of care patients receive within an organization. Topics mentioned in the six approaches include: care that is responsive to the needs and wishes of patients, patient participation in the care process, overcoming communication barriers, providing ‘understandable’ patient information materials, trust, and patients’ rights.

#### Care responsive to needs and wishes

All approaches underline the importance of this issue. The interpretation of this dimension is related to the different visions the approaches have concerning the nature of responsive care:

- CLAS and CRF focus on the *cultural* needs of patients, in accordance with their respective visions on responsive care (‘culturally competent’ and ‘culturally responsive’).
- JCR refers to *‘additional’* and *‘unique’* needs that should be integrated in the clinical process: “it is important for hospitals to be prepared to identify and address not just the clinical aspects of care, but also the spectrum of each patient’s demographic and personal characteristics”.
- COER focuses on the needs of *migrants* (broadly defined)*,* going beyond cultural factors to consider social position, migration history and legal situation.
- EDS and EQS focus on needs resulting from patients’ *individual* characteristics.

Apart from identifying needs, JCR also discusses the points in the care continuum at which they should be taken into account. Although all approaches emphasize the importance of taking patients’ needs and wishes into account, they leave it up to the professional to reconcile the demands that patients or their relatives may make with the dictates of their professional conscience.

#### Patient participation in the care process

Five approaches (JCR, CRF, COER, EDS) explicitly refer to the importance of patient participation or involvement in the individual care process, for example in shared decision making about treatment and care planning [27]. CLAS and EQS do not refer explicitly to patient participation in this context; however, the standards they provide show that they too consider patients as active participants in their treatment.

#### Overcoming communication barriers in patient-provider contact

All approaches except EDS emphasize that organizations should systematically tackle language barriers in the service delivery setting, placing the onus on the organization to provide patients with language assistance where necessary. Various types of interpreting are recommended such as professional interpreters, bilingual staff or intercultural mediators; approaches differ according to which type they prefer. CLAS, JCR and COER explicitly advise against using untrained, informal interpreters such as family members. CLAS, JCR, CRF and EQS assert that organizations are responsible for ensuring the quality and competence of language assistance that is offered. CLAS and JCR mention that patients should be informed about their right to language assistance. JCR and EQS also discuss support to patients with other communication barriers (e.g. hearing or speech impairments).

#### Understandable patient information materials

With the exception of EDS, all approaches stress that patient information materials should be understandable for all patients, in terms of both language and level of health literacy. When suitable materials are not available, CLAS asserts that patients have a right to orally translated information. These points concern not only patient folders providing information about specific medical problems or treatments, but also consent forms and labeling of medicines.

#### Trust

The approaches discuss several issues related to building trust between users and service providers. The first of these is related to the environment within the health care organization: CLAS, JCR and EQS stress the importance of making this welcoming and inclusive. Some approaches include statements underlining the security of patients, stressing that patients should not be exposed to any dangers and mistreatment that might arise from their vulnerability. Phrases used include “patients are free from abuse, harassment, bullying, violence” (EDS); and “the patient has the right to be free from neglect, exploitation, and verbal, mental, physical and sexual abuse” (JC Requirements (see Table 1), p.54 [20]).

A second issue related to trust concerns conflict and grievance procedures. CLAS and JCR recommend that these procedures should be capable of identifying issues that concern organizations’ responsiveness to diversity, and that such conflicts should be dealt with in a respectful manner (CLAS, JCR, and EDS). The issue of access by minority patients to grievance procedures is also discussed (CLAS, JCR), including the need for personnel dealing with complaints and grievances to receive proper training (CLAS).

#### Patients’ rights

CLAS and JCR discuss the importance of informing patients about their rights. This concerns (among other things) the right in the US to receive language assistance (CLAS, JCR) and not to experience discrimination (JCR). JCR and EQS also note the importance of adapting informed consent procedures to the patients’ needs (e.g. health literacy level).

### Patient and community participation at organizational level

The sixth dimension identified in the approaches concerns the involvement of users and communities in health care at the organizational level. In this domain one dimension appeared: involving patients *in the development of services.* Patient participation *in the care process* was subsumed under the previous domain.

#### Involving patients and communities in the development of services

The issue of participation at the organizational level is discussed by all approaches. The first argument put forward in favor of such participation is that it results in more responsive care (CLAS, JCR). Another advantage named is that patients and communities can contribute to the implementation of changes (EDS). The approaches explicitly (CLAS, CRF, COER, EDS, EQS) or implicitly (JCR) assume that their target populations often belong to disadvantaged groups that may normally be less likely to take part in participation processes. The approaches therefore pay attention to the challenge of creating inclusive participation processes.

Four approaches explicitly mention community as well as patient participation (CLAS, JCR, CRF, EDS; COER speaks of migrant participation). The important difference between patient (or user) and community participation is that the latter brings in the voice of people who did not get into treatment. However, only CLAS and EDS explicitly regard it as important to build partnerships (e.g. with community representatives or organizations) in the community served by the health care organization. Their argument amounts to the following: a health care organization serves a community; therefore the community has to be enabled to exert influence on what happens in the organization through a collaborative process. In the other approaches, patients and community members are regarded as complementing each other (usually in the same sentence), without making clear the additional value of community participation.

### Promoting responsiveness

Issues were classified in this domain if they concerned the promotion of responsive health care in the wider society. We identified one dimension, ‘sharing information on experiences’ in improving care for ethnic minority patients.

#### Sharing information on experiences

All approaches except CRF mention the importance of sharing experiences in promoting responsiveness. This is proposed with different aims in mind: to increase support for responsive care from the general public (COER), to demonstrate an organization’s commitment (CLAS, JCR), or to enable organizations to learn from each other (CLAS, EQS). CRF and EQS take this theme a step further, by proposing that organizations should enter into active partnerships with others that promote equity within the health care system (e.g. in research and other collaborative activities).

### Unique issues

Two issues were unique to particular approaches. JCR repeatedly mentioned the identification and addressing of patients’ mobility needs (e.g. using a cane, guide dogs). EDS emphasized “supporting the workforce to remain healthy”, which is in line with its focus on equality in the workforce.

#### ‘Vertical’ analysis (comparing approaches)

In the foregoing section we discussed findings in terms of the domains (the rows of the matrix in Table 3). In what follows we analyze differences between the approaches represented in the columns, to obtain insight into the specific nature of each approach.

**Table 3:** The approaches inserted in the analytic framework

DOMAINS & dimensions						
Organizational Commitment	CLAS	JCR	CRF	COER	EDS	EQS
Policy and leadership	*a written strategic plan to provide culturally competent care *strategic plan is integrally tied to the organization's mission, principles, service focus	*demonstrate leadership commitment to effective communication (EC), cultural competence (CC), and patient- and family centered care (PFCC) *integrate concepts of EC, CC, PFCC into existing policies	*implement a Cultural Responsiveness (CR)-plan addressing the standards *integrate CR-plan into existing services’ plans and processes *demonstrate leadership *have an advising structure with participation of culturally and linguistically diverse (CALD) populations *allocation of financial resources *employ a cultural diversity staff member when CALD population > 20 %	*organization as a whole must be ‘culturally competent’ *implement the recommendations in a sustainable, coordinated and evidence based way	*leaders conduct and plan business so that equality is advanced *managers support and motivate staff to work culturally competent *recruit, develop and support strategic leaders to advance equality outcomes *integrate equality objectives into mainstream business planning	*a specific plan to promote equity, integrated with existing accountability systems *all organization plans promote equity
Measuring and improving performance	*initial and on-going self-assessment of CLAS-related care *integrate CLAS-related measures into regular quality improvement programs (e.g. internal audits) *use data on individual patients for needs assessment, service planning and quality improvement	*a baseline assessment whether organization meets unique patient needs	*obligatory reporting on CR performance (on defined measures) *perform research in outcomes (e.g. emergency presentations) for CALD patients’ care needs *analyze quality/risk management data for CALD patients *report on CR performance in organization's regular performance reports *include CALD stakeholders in performance review	*evaluate existing services, identify existing problems, develop good practices *conduct research to identify problems, determine actions and evaluate interventions	*analyze performance, agree (with stakeholders) on results, and prepare equality objectives	*continually identify and monitor access and barriers in access, and evaluate interventions for reducing access barriers (e.g. communication support services) *use data on equity performance to improve equity in accessibility and quality
Collecting data	CLAS	JCR	CRF	COER	EDS	EQS
Data *on the population at large*	*maintain a current demographic, cultural and epidemiological profile, and a needs assessment of the community	*use available population-level demographic data of surrounding community	*monitor community profile and demographics	*governments (in partnership with other relevant organizations) collect background data and epidemiological data on migrants	*assemble evidence drawing on existing information systems (incl data *on population level*)	*collect or have access to data on health status and health inequalities of catchment area
Data *on the patient population*	*collect data on individual patient's race, ethnicity, spoken/written language in health record *integrate CLAS-related measures into patient satisfaction assessments	*develop a system to collect patient-level data *collect data on patient race and ethnicity in medical record *collect data on patient's language and additional patient-level information (e.g. cultural, religious) *Collect feedback from patients, families	*develop appropriate information strategies for data collection, reporting and sharing *collect CALD patient satisfaction data		*assemble evidence including surveys of patient experiences	*organization's systems can measure equity performance *identify patients' needs according to characteristics (health records include information on demographic characteristics e.g. language, health literacy level, ethnicity)
Staff/workforce	CLAS	JCR	CRF	COER	EDS	EQS
Staff competencies	*all staff receive on-going education in providing CLAS	*new and existing training addresses issues of EC, CC, PFCC	*provide staff at all levels with opportunities to enhance their CR *train staff *CR references included in HRM policies and practices (e.g. position description) *communication systems for sharing information on CR	*care professionals at various levels should be trained in accessibility issues and in cultural competence	*enable staff to be confident and provide appropriate care with support by training, personal development and performance appraisal	*all staff is aware and competent to address inequities due to education *specific training on equity issues *include equity issues in organization's core education
Diversity in workforce	*strategies to recruit, retain and promote diverse staff, representative of demographic characteristics of service area *diverse staff at all levels, including diverse leadership	*recruitment efforts to increase a diverse workforce that reflects the patient population *diverse workforce can increase ethnic and language concordance, which may improve communication		*recruitment policies should ensure that the diversity of general population is reflected in the workforce *(mentioned as an example)*	*fair selection processes to increase diversity of all workforce *equality in levels of pay *staff can work in a safe environment (e.g. free from abuse, harassment etc.) *flexible working options	*fair and equitable workforce policies and practices *promote respect for dignity of all staff and volunteers
Ensuring access	CLAS	JCR	CRF	COER	EDS	EQS
Entitlement to care				*legislation concerning entitlement is properly implemented *professionals at all levels are aware of eligibility rights		*monitor situations of people that are ineligible for care *ensure health care to people ineligible for services by providing appropriate support
‘Understandable' information	*patient related materials and post signage essential for access should be made easily understood *offer and provide language assistance services to all patients with LEP, at all contacts, in a timely manner during all hours of operation			*programs for migrants should include knowledge on health and illness, the way the health system works, and entitlements to health services *promote interpretation and translated materials to improve accessibility		*in communicating with people and providing information on access issues, health literacy and language needs are taken into account
Geographical accessibility				*inconvenient locations should be reduced as far as possible		*minimize architectural, environmental and geographical barriers to facilities
Other aspects of accessibility				*remove accessibility barriers and reduce practical difficulties (e.g. inconvenient opening times)	*patients, carers and communities can readily access services *public health, vaccination and screening programs benefit all local communities/groups	*ensure access to care for disadvantaged people *provide outreach information to disadvantaged people on outreach services
Care provision	CLAS	JCR	CRF	COER	EDS	EQS
Care responsive to needs and wishes	*patients receive effective (positive outcomes), understandable and respectful (patients values taken into account) care *care should be compatible with cultural health beliefs and practices, and preferred languages	Throughout the care continuum: *ask for additional needs (e.g. cultural, religious) *communicate information about unique patient needs to relevant providers *start patient-provider interaction with an introduction *identify and accommodate cultural, religious, spiritual beliefs/practices that influence care *incorporate EC, CC, PFCC concepts into care delivery	*inclusive practice in care planning (including dietary, spiritual and other cultural practices) *implementation and revision of policies for provision of appropriate meals *use feedback/evaluation from patients to improve CR *develop and use suitable instruments for assessment (e.g. clinical diagnosis) which incorporate cultural considerations	*improve relevance and appropriateness of health services *offering the same services to everybody may result in users receiving lower quality of care *services should be culturally competent (matched to needs of migrants from diverse backgrounds) *culturally competent care goes beyond cultural factors, e.g. social position, history, legal situation should also be taken into account *adapt existing diagnostic and therapeutic procedures or invent new ones if necessary	*assess individual patients' health needs and provide appropriate and effective services *discuss changes across services with patients and make transitions smoothly *strive for positive treatment experiences: being listened to, being respected, privacy and dignity are prioritized	*In needs assessments, delivery of care and at discharge, patients’ individual, family characteristics, experiences and living conditions are taken into account (incl. psychosocial needs) *workforce is able to take into account individual patients' ideas and experiences of health and illness in clinical practice and at discharge *care is considerate and respectful of patients' dignity, personal values, knowledge and beliefs regarding health care
Patient participation in the care process		*Involve patients, families, support persons in the care process along the care continuum.	*inclusive practice in care planning (including dietary, spiritual and other cultural practices)	*promote participation of migrants in all activities concerning the provision of health services, including decision making processes	*involve patients as they wish during the care continuum	
Overcoming communication barriers in patient-provider contact	*offer and provide language assistance services (including bilingual staff, interpreter services) at no costs to all patients with LEP, at all contacts, in a timely manner during all hours of operation *inform patients of their right to receive language assistance *assure competence of language assistance by interpreters and bilingual staff	*identify patient's preferred language or other communication needs during admission *identify and monitor patient communication needs/status during care continuum, document this in patient record *ensure competence of language assistance *develop a system to provide language services *inform patients of their rights for an interpreter	*implement language services policy *policies include directions about role of interpreters and family *provide accredited interpreters to patients who need one *match employment of in-house interpreters to community demographics *evaluate interpreter services	*high quality interpreting should be promoted *consider all available methods to reduce language barriers		*have a policy on overcoming language barriers *make professional interpreting services available and promote it *accommodate communication needs of patients with e.g. hearing, speech impairments *monitor quality of interpreting services/communication support *ensure staff ability to work with interpreter/communication support staff
‘Understandable’ patient information materials	*provide easily understood patient related materials (applications, consent forms) and post signage in diverse languages incl. directions to facility services *(diverse language: languages of commonly encountered groups/groups represented in the service area)* *take into account culture and health literacy levels *persons from small language groups have the right to oral translation	*provide patient education materials and instructions that meet patients' needs (health literacy, language) during assessment, treatment and discharge *support patient’s ability to understand/act on health information *determine needs for assistance with admission forms (health literacy)	*have appropriate translations of signage, patient forms, education materials for predominant language groups using services	*promote high quality translated written information		*provide easily understood written material and signage taking health literacy and language needs into account
Trust	*Conflict & grievance* *conflict/grievance procedures are culturally sensitive *conflict/grievance procedures can identify, prevent, resolve cross-cultural conflicts/complaints *staff handling complaints should receive cultural competence training *monitor culturally or linguistically related complaints *Atmosphere* *create a welcoming and inclusive environment	*Conflict & grievance* *accessible complaints system (language, non-writing) *complaints are not being subjected to coercion, discrimination, reprisal, or unreasonable interruption of care *Atmosphere* *create an environment that is inclusive of all patients *patient has the right to be free of neglect, exploitation and abuse *(regular JC standards, chapter: Rights and Responsibilities of the Individual)*	*Conflict & grievance* *monitor number of complaints lodged by CALD consumers/patients.		*Conflict & grievance* *complaints should be handled respectfully and efficiently *Atmosphere* *create a safe environment, without threat of dignity of denial of individual identity	*Atmosphere* *create a safe environment, with respect for patient's dignity and identity *create an environment inclusive for all patients
Patients’ rights	*provide notices in diverse language of a variety of patients’ rights (including right for language assistance)	*inform patients of their rights (interpreter, accommodation for disability, be free from discrimination, etc.) *tailor the informed consent process to meet patient needs (related to low HL)				*accommodate patients' diverse needs in informed consent procedure
Patient and community participation at organizational level	CLAS	JCR	CRF	COER	EDS	EQS
Involving patients and communities in the development of services	*utilize a variety mechanisms to facilitate community and patient involvement in designing and implementing services *develop participatory, collaborative partnerships with communities	*be involved and engaged with patients, families and the community to identify needs for new/modified services *collect feedback from patient, families and communities	*CALD consumer, carer and community members are involved in the planning, improvement and review of programs and services on an on-going basis *advice of participation structures is taken into account *facilitate different degrees of participation from CALD consumers, carers, community *develop partnerships with ethno-specific community organizations	*promote participation of migrants in designing, evaluating, and carrying out research on migrant health and health care *promote participation of migrants in developing and implementing new measures	*identify local interests (including patients, communities) that need to be involved in implementing EDS *share assembled information with local interests so they participate in analyzing performance and setting objectives *agree roles with local authority (e.g. services that share the same clientele)	*identify service users at risk for exclusion from participatory processes, promote their participation *identify and overcome barriers to participation *monitor and evaluate participatory processes *use feedback to improve services and share results of participation with public
Promoting Responsiveness	CLAS	JCR	CRF	COER	EDS	EQS
Sharing information on experiences	*make information available to public on progress and innovations in implementing CLAS *inform community, own organization (for institutionalizing CLAS) and other organizations to learn from each other	*share information with surrounding community about efforts to meet unique patients’ needs to demonstrate commitment	*undertake research to develop new and improved initiatives and resources for CR	*inform public adequately about issues concerning migrant health	*share assembled evidence on equality performance with local interests (e.g. patients, communities), so they can take part in analysing performance and set goals *publish accomplishments (grades) and equality objectives so they are accessible for local interests	*be a participant in networks, research initiatives which promote equity *disseminate results of research/practice related to equity *build solid relationships/ networks with community-based service providers *ensure that equity is reflected in all partnership and service contracts
Unique issues	CLAS	JCR	CRF	COER	EDS	EQS
		*identify and address mobility needs (e.g. cane, guiding dogs)			*support workforce to remain healthy, focus on major health and lifestyle issues that affect individual and wider population	

The linguistic issues in CLAS have a legal basis in the Civil Rights Act of 1964, which requires “entities that receive Federal financial assistance to take steps to ensure that limited English proficient individuals have meaningful access to the health services” [9], p. 10). CLAS has a very explicit vision on responsive health care, for example in the details provided about the content of training or the types of data that should be collected. Although unequal access is mentioned as a problem which might be reduced by providing appropriate services, the CLAS standards discuss only linguistic barriers to access.

JCR follows the steps in the clinical care process, which is explained by its origin in the Joint Commission standards for hospitals. JCR was developed as a supplement to existing standards, so it may have left out some issues already covered by existing Joint Commission standards. The standards do not embody an elaborated vision on ‘responsiveness’ beyond cultural competence, communication, and patient- and family-centered care. Accessibility is not operationalized in JCR. Interestingly, the international branch of the Joint Commission does consider accessibility as an important aspect of health care, as demonstrated for example by the standard stating that “the organization seeks to reduce physical, language, cultural, and other barriers to access and delivery of services” (p.42 [28]).

CRF focuses, like CLAS, mainly on cultural and linguistic issues. CRF offers quantitative and qualitative indicators (standards and measures) for measuring organizational responsiveness to diversity.

COER states to address governments rather than individual health care organizations, by discussing issues concerning organizational responsiveness to diversity without specifying precisely the division of responsibilities between levels. In keeping with the Council of Europe’s historical role, COER is primarily concerned with the ethical and human rights dimensions of social and health issues.

EDS addresses accessibility and quality of care, but its vision of responsive care for ‘protected groups’ remains rather implicit. The implementation strategy elaborates on steps such as ‘engage with local interests’ and ‘analyze performance’. The content of responsive care is only briefly described in terms of goals such as ‘better outcomes for all’ and ‘improved patient access and experience’ (see Table 2). One of the main objectives of EDS was to provide a tool for NHS commissioners to comply with the UK’s ‘Public Sector Equality Duty’ (PSED). This is reflected in the envisaged target groups of EDS and explains the unique focus of EDS on equality among staff, in line with the aim of the PSED to eliminate discrimination and enhance equal opportunities *throughout* the public sector.

EQS emphasizes the vulnerability of certain patients, which can result from many factors – ‘culture’ being hardly mentioned as one of these. The focus is on patients’ individual needs and characteristics, rather than their membership of specific ethnic, cultural or other groups. This approach seems to view ‘patient centered care’ as the best way to respond to diversity in care provision. Although EQS defines its target group as ‘migrants and other vulnerable groups’, most of the standards focus on issues relevant to migrants, which may be explained by the Task Force’s origin in the Migrant Friendly Hospital network [25].

#### Variations in the orientation of different approaches

Only the European approaches address issues of access to health care in the sense of entitlement. A common feature of the non-European approaches is their emphasis on ‘culture’. On closer examination, this seems to be mainly a question of how factors are labeled: in CLAS, for example, differences in socioeconomic or legal status are regarded as ‘cultural’ ones. In the European approaches the focus is on individual characteristics, which brings EQS close to the approach known as ‘patient centered care’ [29] (Saha, Beach, and Cooper have discussed the relation between ‘patient centered’ and ‘culturally competent’ care [30]). A possible shortcoming of this individualistic perspective is that the social position that characterizes members of certain groups (e.g. asylum seekers, irregular migrants) may be overlooked. COER and EQS are the only approaches that explicitly refer to ‘migrants’ and take into account the vulnerability that results from having different kinds of migrant status (asylum seeker, irregular migrant, labor migrant etc.).

## Discussion

From this content analysis of six approaches to organizational responsiveness to diversity a broad consensus emerged on what health care organizations need to do to meet the differing health care needs of diverse patient populations. The following domains were almost universally regarded as important for creating responsive organizations: organizational commitment, collecting and analyzing data to provide empirical evidence on inequalities and needs, development of a competent and diverse workforce, ensuring access for all users, ensuring responsiveness in care provision, fostering patient and community participation, and actively promoting the ideal of responsiveness (see Tables 3 and 4 for the synthesis of the content of these domains).

**Table 4:** Description of classified domains and dimensions, and coverage of domains/dimensions by the six approaches (the orange cells visualize table 3’s empty cells meaning that this dimension is not covered by that approach)

	

The described elements for organizations to address might be used as guidance for implementation of diversity responsive health care. The elements show that all stakeholders, including all patients and their communities, should be involved in developing improved services. This consensus opens the way to wider implementation of organizational responsiveness to diversity.

There were, however, also some variations in the operationalization of diversity responsive health care, related to different scopes, contexts and types or definitions of diversity. The non-European approaches focus on improving quality of care and linguistic accessibility in the fight against health disparities, while the issue of ‘entitlement to care’ seems overlooked. This is remarkable since (lack of) health insurance coverage explains substantial proportions of disparities in health care in the US [31]. The National Health Interview Study 2012 found that while 7.6 % of white respondents had been uninsured for more than one year, this percentage was 23.6 in the Hispanic population, 11.7 in the black population, and 11.3 in the Asian population [32]. Although entitlement to care is usually regulated by governments rather than service providers, approaches that aim to reduce disparities should at least acknowledge the importance of entitlement.

The consensus we found between these six approaches relates to some emerging debates in the area of diversity responsive care. First, there is the debate on the relative importance of individual patient characteristics versus cultural or group characteristics. Adapting routine health and preventive care services [33] using the concept of ‘patient centered care’ is a step in the right direction of acknowledging diversity among all patients. However, patient centeredness focuses on acknowledging the uniqueness of patients. This individualistic approach to diversity carries a risk, because the most serious inequities in health care are strongly associated with differences in group membership and social situation. This was illustrated in Philimore’s study on maternity services in an era of superdiversity, which showed that immigration status was one of the most important factors in determining problems with maternity services [34]. Being an asylum seeker or undocumented migrant is neither a cultural characteristic nor a personal one – it is a social position, with important consequences for health and access to health care. An exclusive on ‘culture’ is misleading, and that diversity responsive health care means that cultural aspects, social position and individual needs all have to be taken into account.

A second debate touching the topic of this paper is the debate of discrimination versus diversity responsive health care. The latter explicitly requires care providers to differentiate between patients (if relevant for equal chances of optimal health care outcomes), whereas the non-discrimination principle prescribes not making a distinction between patients (under equal circumstances) [35]. Dovidio et al. report that overt racism among care providers has become rare, but that explicit egalitarian attitudes prevent care providers and organizations to diversify care provision when it is necessary [36]. The fear of prejudice and stereotyping may thus lead to suboptimal care. The topic of discrimination was only briefly touched upon in most of the six analyzed approaches. We believe that future approaches should elaborate more on this topic so that health care organizations will have more guidance in dealing with this dilemma.

Our analysis had its limitations. The sample of approaches was confined to the US, Australia and Europe. We explicitly did not aim at a complete review by sampling and comparing all existing approaches, because too many have been developed to make that practical. It is possible that valuable approaches were missed. However, the six approaches included showed considerable consensus regarding the important elements of care that is responsive to diversity, and data were saturated. Because of variations in the level of detail provided by the approaches, classifying them may not fully do justice to the visions or ideas behind the approaches. For example, EDS contains only a rudimentary conceptualization of responsiveness, but it may be that within the NHS other documents elaborate this concept in more detail.

## Conclusion

This study demonstrated that a considerable degree of consensus exists about the issues that health care organizations need to address in order to adequately respond to diversity. This creates the opportunity to move forward, to resolve purely terminological differences and to help health care organizations to respond to the diversity that is present in modern societies.

## Abbreviations

CLAS: Culturally and Linguistically Appropriate Services (referring to the National Standards for Culturally and Linguistically Appropriate Services in Health Care
JCR: Joint Commission Roadmap (referring to Advancing Effective Communication, Cultural Competence, and Patient- and Family Centered Care: A Roadmap for Hospitals
CRF: Cultural Responsiveness Framework (referring to Cultural Responsiveness Framework. Guidelines for Victorian health services
COER: Council of Europe Recommendations (referring to Recommendation of the committee of ministers to member states on mobility, migration and access to health care
EDS: The Equality Delivery System
NHS: National Health Service
EQS: The Equity Standards (referring to Standards for Equity in Health Care for Migrants and other Vulnerable Groups
WHO-HPH: World Health Organization - Health Promoting Hospitals Network
HRM: Human Resource Management
PSED: Public Sector Equality Duty
